# Supplementary material for: The extrafollicular B cell response is a hallmark of childhood idiopathic nephrotic syndrome
Source: Nat Commun. 2023 Nov 24;14:7682. doi: 10.1038/s41467-023-43504-8 (PMC10667257; doi:10.1038/s41467-023-43504-8)
Supplement: Supplementary file 1 — Supplementary Information [file 41467_2023_43504_MOESM1_ESM.pdf]

Supplementary Information

Title: The extrafollicular B cell response is a hallmark of childhood idiopathic nephrotic syndrome.

Author list: Tho-Alfakar Al-Aubodah[1,2,3,4,5], Lamine Aoudjit[3,5], Giuseppe Pascale[5,6], Maneka A. Perinpanayagam[7], David Langlais[1,8], Martin Bitzan[6,9], Susan M. Samuel[7], Ciriaco A. Piccirillo[1,2,4]*, Tomoko Takano[3,4,5]*

[1] Department of Microbiology & Immunology, Faculty of Medicine and Health Sciences, McGill University, Montréal, Québec
[2] Infectious Diseases and Immunity in Global Health Program, Research Institute of the McGill University Health Centre, Montréal, Québec
[3] Metabolic Disorders and Complications Program, Research Institute of the McGill University Health Centre, Montréal, Québec
[4] Centre of Excellence in Translational Immunology, Research Institute of the McGill University Health Centre, Montréal, Québec
[5] Division of Nephrology, Faculty of Medicine and Health Sciences, McGill University, Montréal, Québec
[6] Division of Nephrology, Department of Pediatrics, Faculty of Medicine and Health Sciences, McGill University, Montréal, Québec
[7] Section of Nephrology, Department of Pediatrics, Cumming School of Medicine, University of Calgary, Calgary, Alberta
[8] Department of Human Genetics, Faculty of Medicine and Health Sciences, McGill University Genome Centre, Montréal, Québec
[9] Kidney Centre of Excellence, Al Jalila Children's Hospital, and Mohammed Bin Rashid University of Medicine and Health Sciences, Dubai, UAE

*Correspondence should be addressed to:
Dr. Tomoko Takano, M.D., Ph.D.
Research Institute of the McGill University Health Centre (RI-MUHC),
Metabolic Disorders and Complications Program, Division of Nephrology
1001 Boulevard Décarie, Bloc E, Room EM1.3244
Montréal, Québec H4A 3J1, Canada
E-mail: tomoko.takano@mcgill.ca

Dr. Ciriaco A. Piccirillo, Ph.D.
Research Institute of the McGill University Health Centre (RI-MUHC),
Infectious Diseases and Immunity in Global Health Program
1001 Boulevard Décarie, Bloc E, Room EM2.3248
Montréal, Québec H4A 3J1, Canada
E-mail: ciro.piccirillo@mcgill.ca

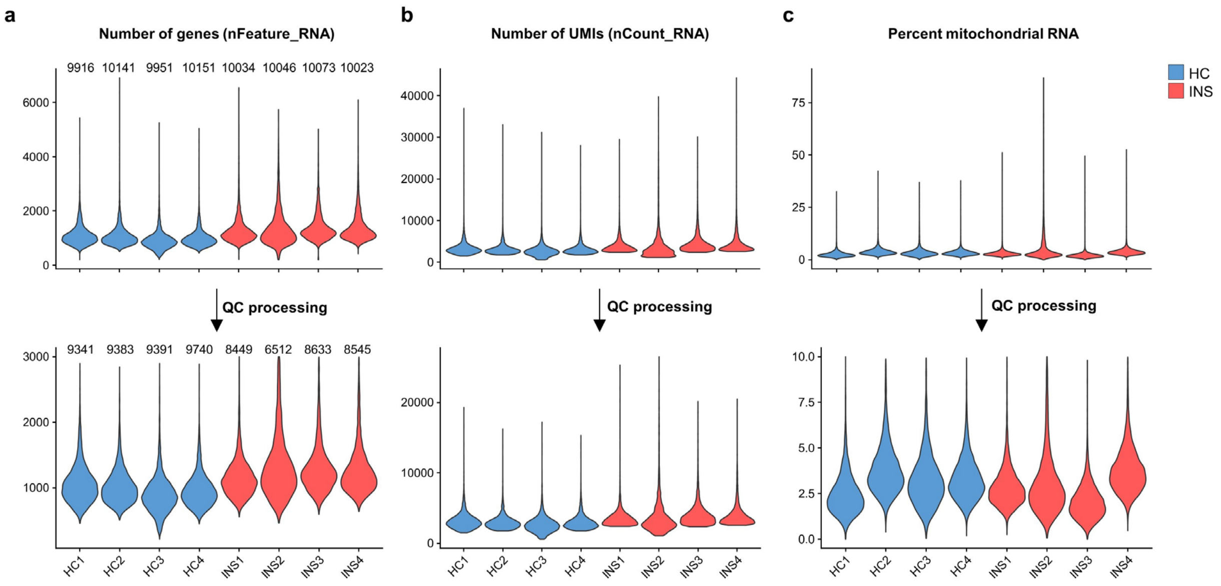

Supplementary Figure 1: Quality control processing of HC and INS PBMC from scRNA-seq. a.-c. The number of genes (nFeature_RNA) (**a**), UMIs (nCount_RNA) (**b**), and frequencies of mitochondrial RNA (**c**) in each sample before (*top row*) and after (*bottom row*) quality control (QC) processing. QC steps are outlined in the methodology. This Supplementary Figure is associated with Figure 1.

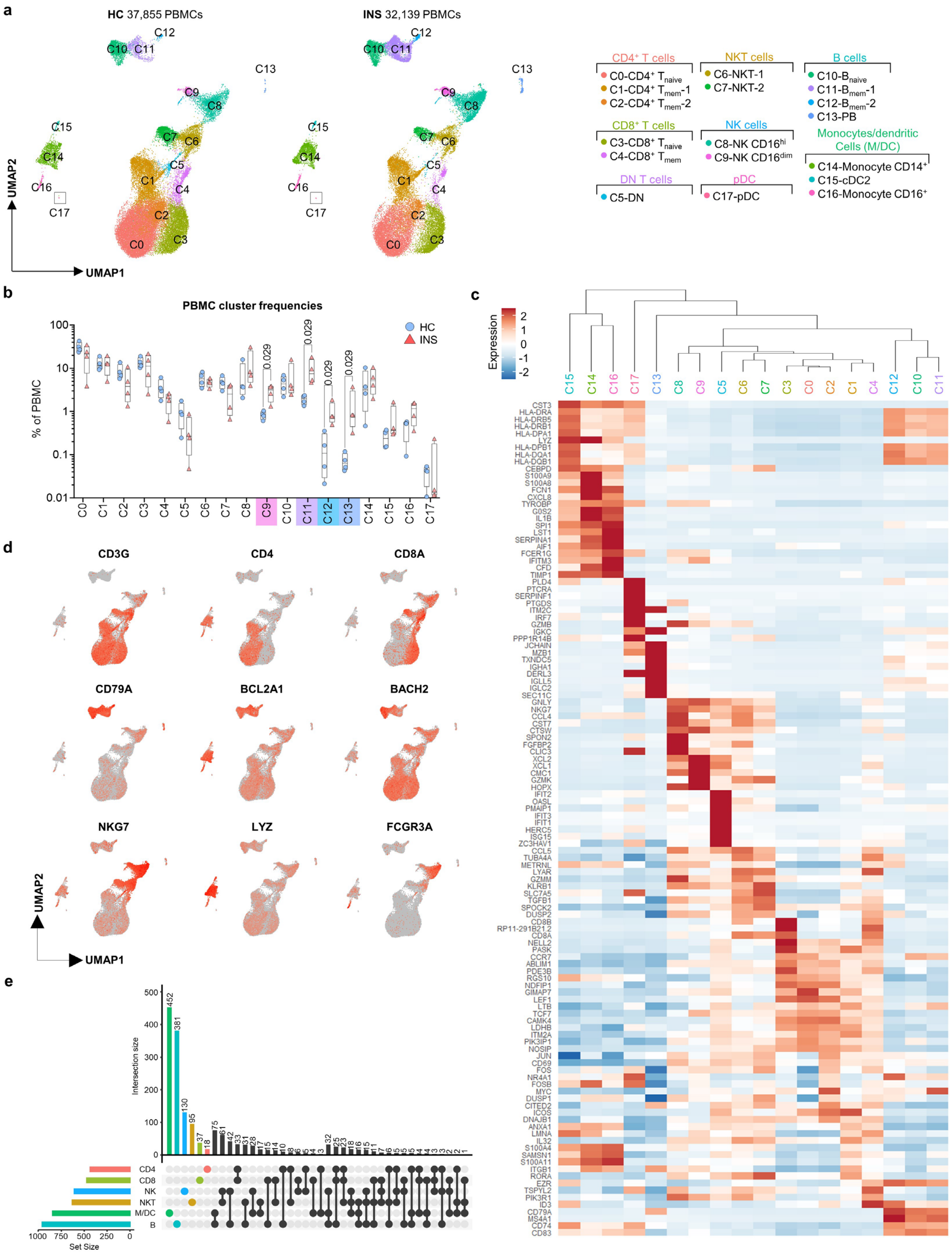
a
HC 37,855 PBMCs
INS 32,139 PBMCs
UMAP2
UMAP1
CD4+ T cells
C0-CD4+ T naive
C1-CD4+ T mem-1
C2-CD4+ T mem-2
NKT cells
C6-NKT-1
C7-NKT-2
B cells
C10-B naive
C11-B mem-1
C12-B mem-2
C13-PB
CD8+ T cells
C3-CD8+ T naive
C4-CD8+ T mem
NK cells
C8-NK CD16hi
C9-NK CD16dim
Monocytes/dendritic Cells (M/DC)
C14-Monocyte CD14+
C15-cDC2
C16-Monocyte CD16+
DN T cells
C5-DN
pDC
C17-pDC
b
PBMC cluster frequencies
% of PBMC
HC
INS
0.029
c
Expression
d
CD3G
CD4
CD8A
CD79A
BCL2A1
BACH2
NKG7
LYZ
FCGR3A
e
Intersection size
CD4
CD8
NK
NKT
M/DC
B
Set Size

Supplementary Figure 2: Annotation of PBMC populations by scRNA-seq. a. Integrated Uniform Manifold Approximation and Projection (UMAP) of the 18 clusters of PBMC from HC (*n* = 4) and INS (*n* = 4) children. **b.** Frequencies of each PBMC cluster in HC and INS individuals. Data are shown as box plots depicting the mean (centre), interquartile range (bounds of box), and min-max range (whiskers); each dot corresponds to a single donor (*n* = 4 HC, 4 INS); *P* values were determined using individual two-sided Mann-Whitney U tests. **c.** Heatmap showing the expression of the top ten genes in each PBMC cluster. **d.** Feature plots showing the expression of several major lineage defining genes in PBMC. **e.** Upset plot showing the number of differentially expressed genes (set sizes) between INS and HC within each broad cell lineage along with intersection sizes between gene lists following pseudobulk differential genes expression analysis using the Muscat R package. This Supplementary Figure is associated with Figure 1.

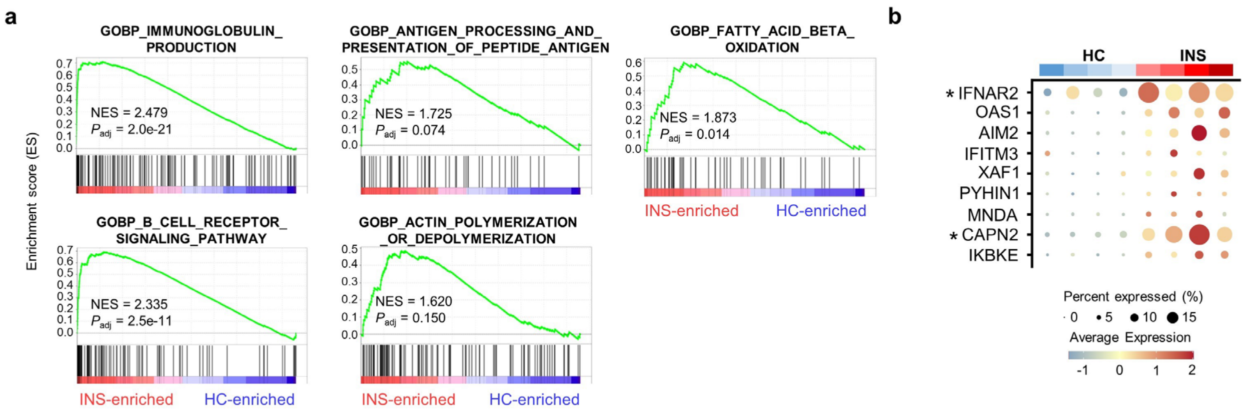

Supplementary Figure 3: Gene set enrichment analysis (GSEA) on the nephrotic B cell signature. a. Results of GSEA of Gene Ontology Biological Processes (GOBP) terms from $\log_2$(Fold Change)-ranked genes following pseudobulk-level differential gene expression analysis between INS and HC B cells. P_{adj} values were determined using the Benjamini-Hochberg correction for multiple-testing. NES, normalized enrichment score. **b.** Bubble plot of highly enriched type-I interferon genes. Asterisks denote genes present in the nephrotic B cell signature. This Supplementary Figure is associated with Figure 1.

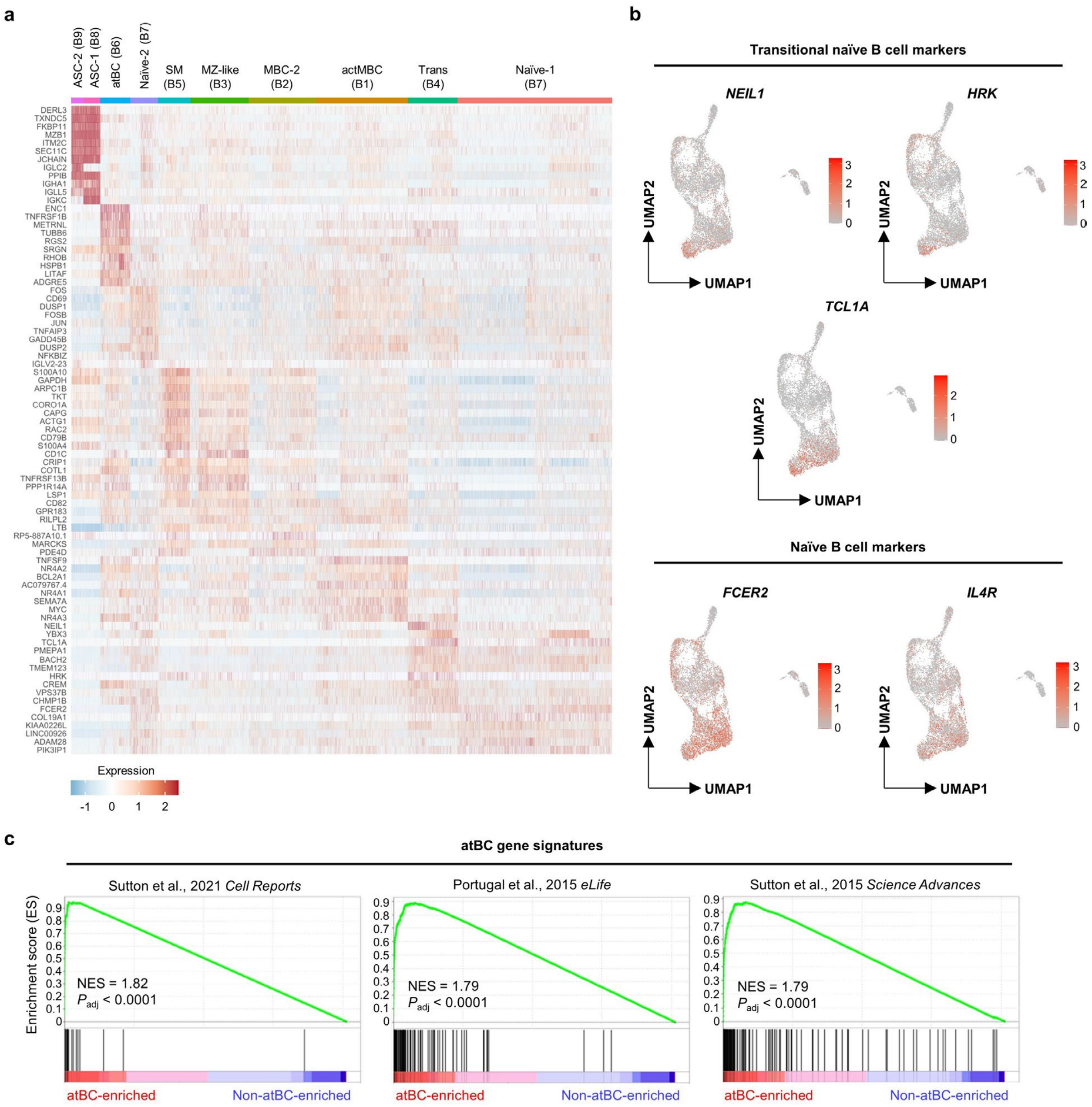

Supplementary Figure 4: a. Heatmap showing the expression of the top 10 genes in each of the B cell subclusters. **b.** Feature plots showing the expression of transitional naïve-associated (*NEIL1*, *HRK*, *TCL1A*) and general naïve-associated (*FCER2*, *IL4R*) genes. **c.** Gene set enrichment analysis (GSEA) plots showing the enrichment of atBC genes from three independent studies with the atBCs identified herein. P_{adj} values were determined using the Benjamini-Hochberg correction for multiple-testing. NES, normalized enrichment score. This Supplementary Figure is associated with Figure 2.

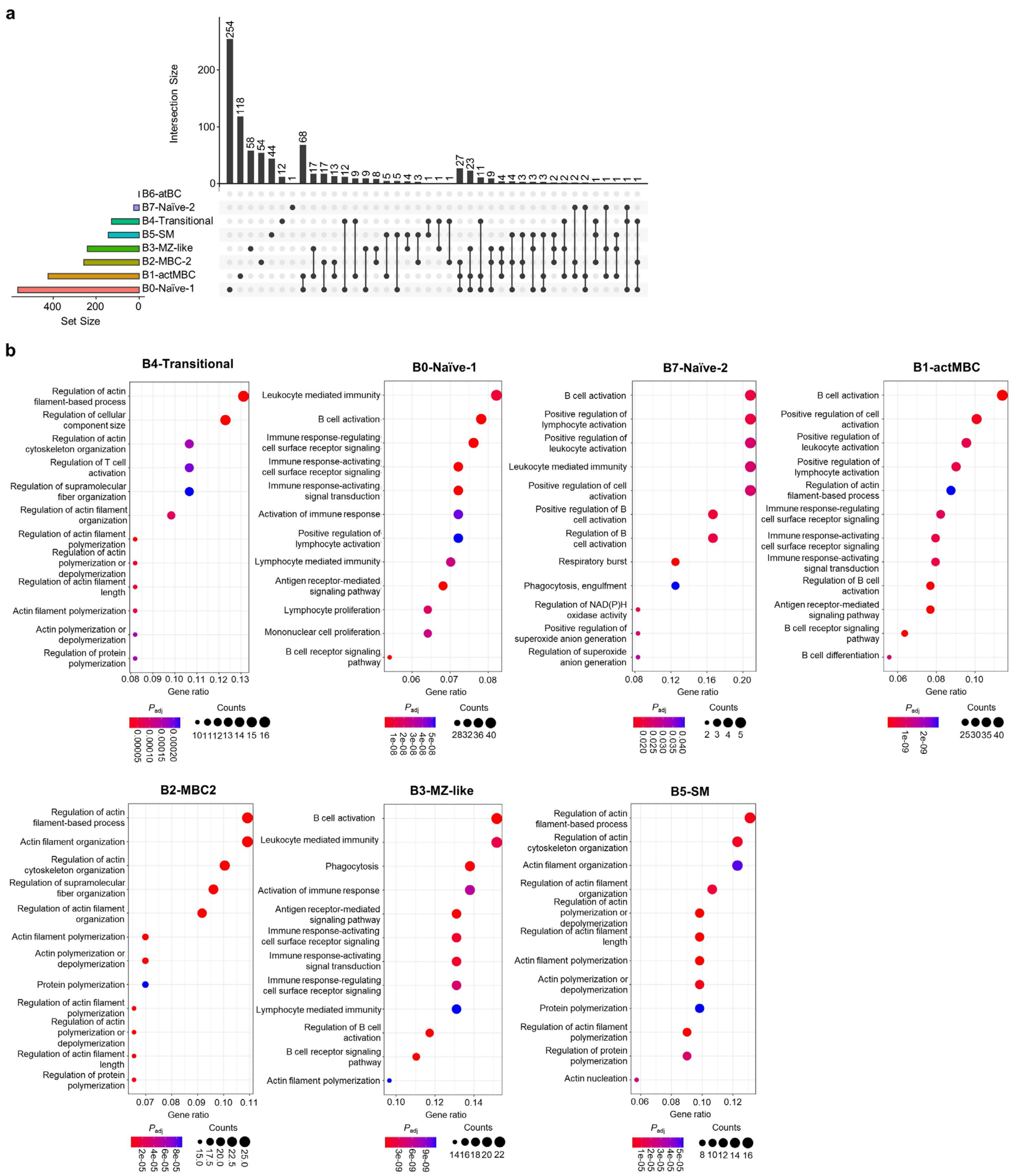

Supplementary Figure 5: Pathway analysis of INS-associated genes in B cell subclusters. a. Upset plot showing the set sizes and intersection sizes of pseudobulk-level differentially expressed genes between INS and HC B cell subclusters using the Muscat R package. **b.** Gene Ontology pathways enriched in INS B cell subclusters over HC B cell subclusters. Genes used for pathway analysis were significantly upregulated (P_{adj}<0.05 and |$\log_2$(Fold Change)|>0.65) in INS versus HC B cell subclusters; P_{adj} values were determined using the Benjamini-Hochberg correction. This Supplementary Figure is associated with Figure 2.

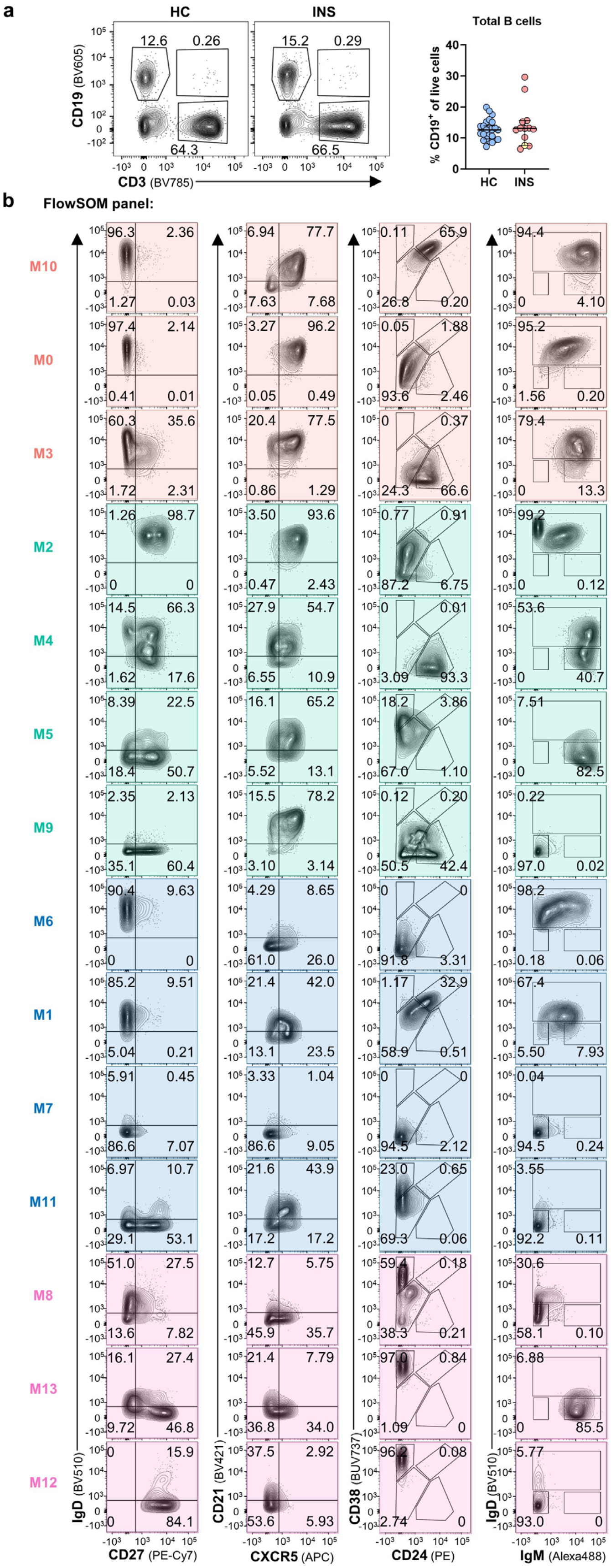
a
HC
INS
Total B cells
CD19 (BV605)
CD3 (BV785)
% CD19+ of live cells
b
FlowSOM panel:
M10
M0
M3
M2
M4
M5
M9
M6
M1
M7
M11
M8
M13
M12
IgD (BV510)
CD27 (PE-Cy7)
CD21 (BV421)
CXCR5 (APC)
CD38 (BUV737)
CD24 (PE)
IgM (Alexa488)

Supplementary Figure 6: Characterization of B cells in HC and INS children using FlowSOM. a. Representative flow plots of live PBMC showing total ($CD19^+$) B cells along with the quantification in HC (n = 24) and INS (n = 13). **b.** Equivalent numbers of randomly selected B cells from HC (n = 24; 195,000 B cells) and INS (n = 13; 195,000 B cells) children were concatenated and used for FlowSOM clustering on the following markers: CD19, CD20, CD21, IgD, IgM, CD27, CD38, CXCR5, CD1c, and CD24. FlowSOM generated 14 metaclusters (M0-M12). The phenotypes of each cluster using traditional gating strategies for B cells are shown. This Supplementary Figure is associated with Figure 3.

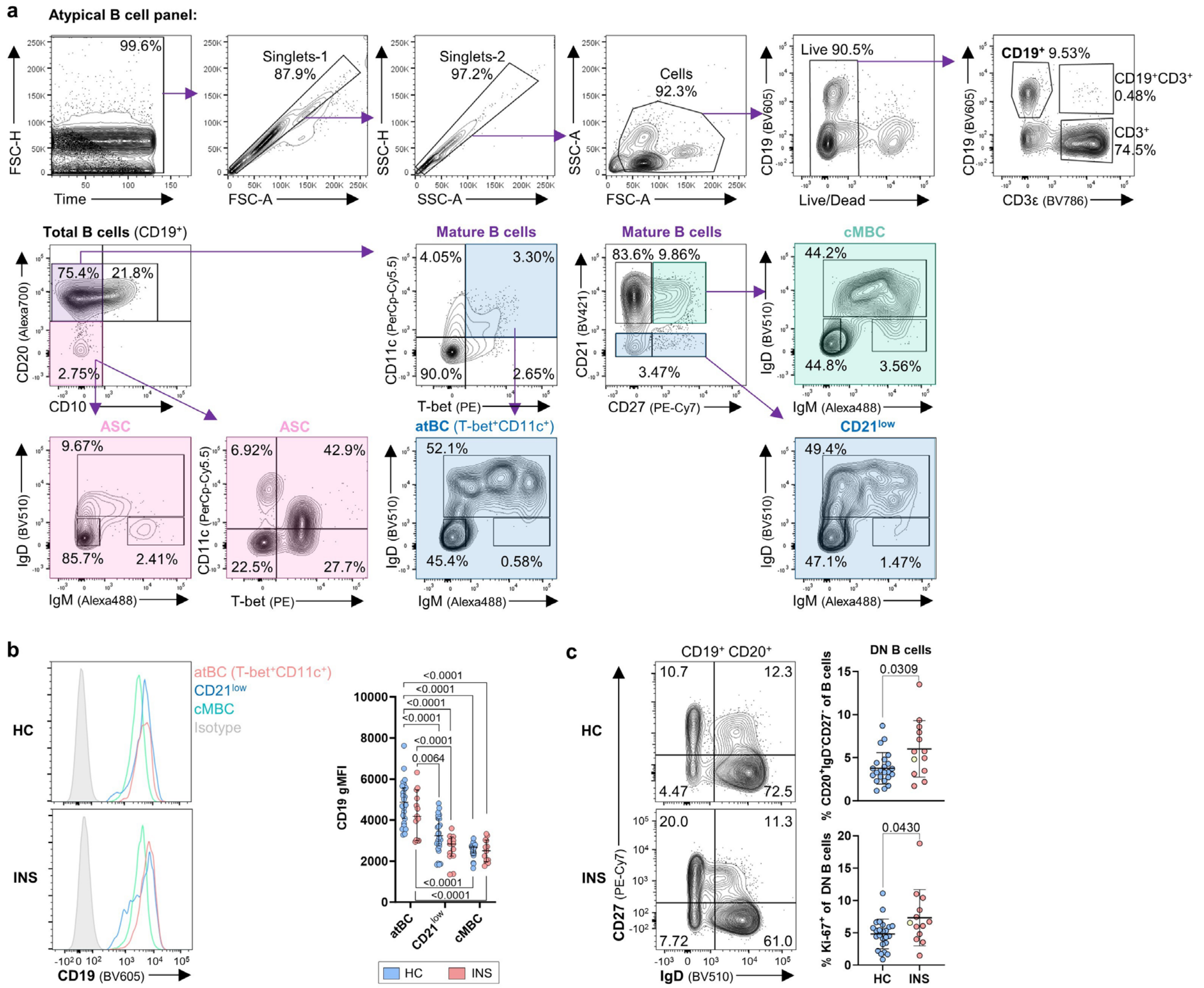

Supplementary Figure 7: Characterization of atypical B cells in HC and INS PBMC. a. Gating strategy for atBCs, cMBCs and ASCs. **b.** Representative histograms and quantification of the expression of CD19 in atBC (T-bet^{+} CD11c^{+} CD21^{low} CD19^{+} CD20^{+} CD10^{-}), CD21^{low} (CD21^{low} CD19^{+} CD20^{+} CD10^{-}), and cMBCs (CD27^{+} CD21^{+} CD19^{+} CD20^{+} CD10^{-}) in HC (n = 24) and INS (n = 13) children. **c.** Representative flow plots and quantification DN B cells (IgD^{-} CD27^{-} CD19^{+} CD20^{+} CD10^{-}) in HC (n = 24) and INS (n = 13) children. Data are shown as median with 95% confidence intervals and P values were determined by two-way ANOVA with Tukey's multiple testing (**b**), or two-sided Mann-Whitney U tests (**c**). Each data point corresponds to a single donor. The yellow data point represents the child with glucocorticoid-resistant membranous nephropathy. atBC, atypical B cells; cMBC, classical memory B cells; ASC, antibody-secreting cells. This Supplementary Figure is associated with Figure 4.

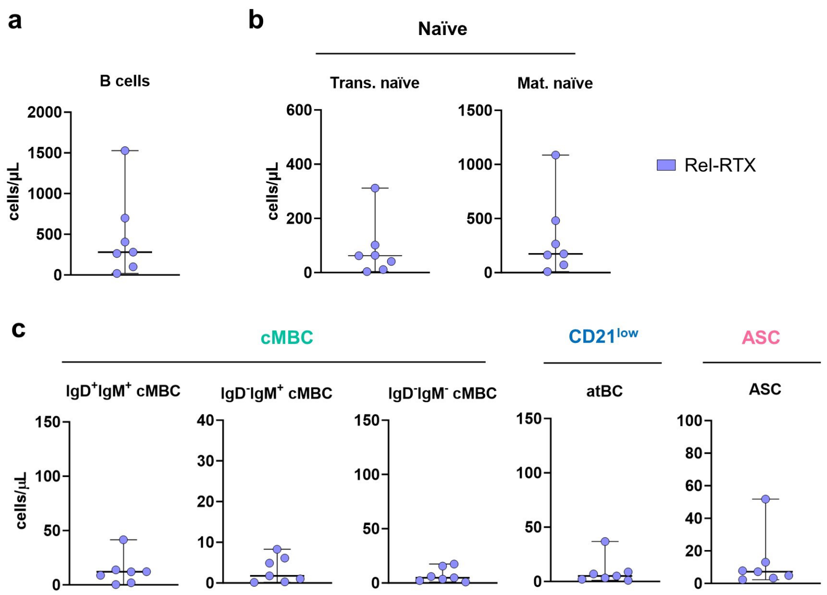

Supplementary Figure 8: Numbers of B cell subsets in relapses following rituximab therapy. a.-c. Absolute numbers of total (**a**), naïve (**b**), and memory (**c**) B cell populations in the PBMC of children in post-rituximab relapse (*n* = 7). Rel-RTX, relapse following rituximab. This Supplementary Figure is associated with Figure 5.

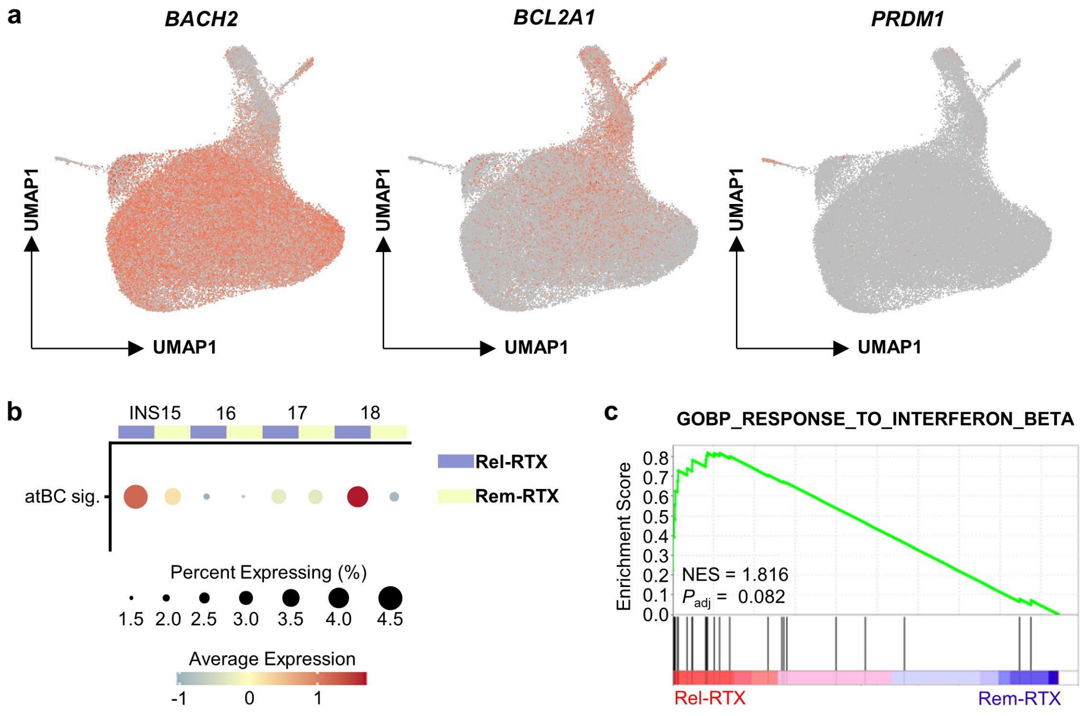

Supplementary Figure 9: scRNA-seq characterization of memory B cells during relapse or remission following rituximab therapy. a. Feature plots showing the expression of *BACH2*, *BCL2A1*, and *PRDM1* on the integrated UMAP clusters of post-rituximab relapse and remission B cells. Rel-RTX, relapse following rituximab; Rem-RTX, remission maintained by rituximab. **b.** Bubble plot showing the module score of the atBC signature in total B cells from each donor during post-RTX relapse and remission. **c.** Gene set enrichment analysis (GSEA) of the Gene Ontology term "Response to Interferon Beta" (GO:0035456) in Rel-RTX memory B cells. P_{adj} value was determined using the Benjamini-Hochberg correction for multiple comparisons. NES, normalized enrichment score. This Supplementary Figure is associated with Figure 6.

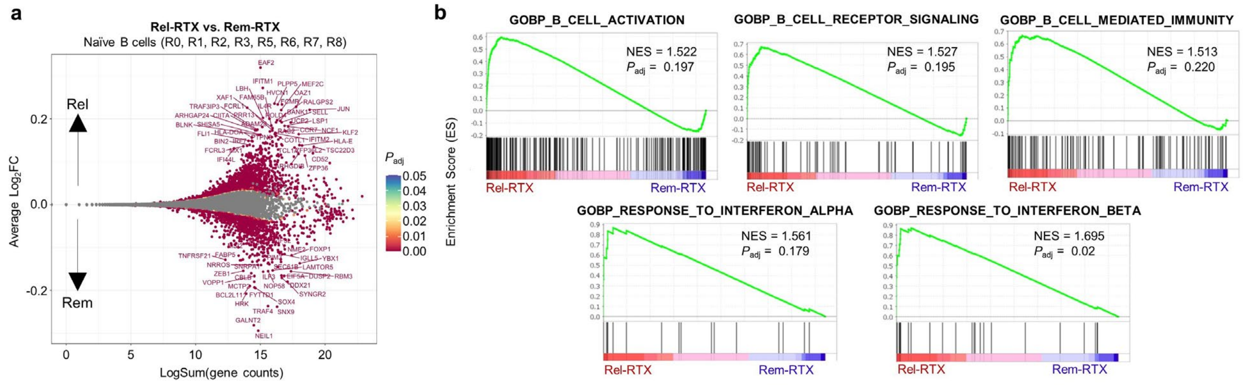

Supplementary Figure 10: scRNA-seq characterization of naïve B cells during relapse or remission following RTX therapy. a. MA plot showing the differential expression of genes within all naïve B cells (subclusters R0, R1, R2, R3, R5, R6, R7, and R8) between Rel-RTX and Rem-RTX. Rel-RTX, relapse following rituximab; Rem-RTX, remission maintained by rituximab. **b.** Gene set enrichment analysis (GSEA) showing the enrichment of various Gene Ontology Biological Processes (GOBP) pathway within the relapse-associated naïve B cells. P_{adj} values were determined using the Benjamini-Hochberg correction for multiple comparisons. NES, normalized enrichment score. This Supplementary Figure is associated with Figure 7.

Supplementary Table 1 – Patient characteristics

	INS	Rel-RTX	Rem-GC	Rem-RTX
Number (M/F)	14 (7/7)	7 (2/5)	13 (6/7)	14 (5/9)
Median age (years, IQR)	8.1 (6.2-10.6)	9.7 (9.1-10.5)	9.9 (7.0-11.9)	9.2 (7.2-10.7)
Median uPCR (g/mmol, IQR)	1.02 (0.47-1.58)	0.75 (0.67-0.79)	0.01 (0.01-0.02)	0.01 (0.01-0.01)
Stage of disease				
First onset	3	0	N/A	N/A
Relapse	11	7	N/A	N/A
Remission	N/A	N/A	13	14
Diagnosis				
SSNS	13	7	13	14
SRNS	1	0	0	0
Biopsy				
MCD	0	2	1	3
FSGS	1	1	0	1
MN	1	0	0	0
Not done	12	4	12	10
Current medication				
Prednisone	3	1	0	0
Tacrolimus	1	1	0	1
MMF	0	0	2	0
Leflunomide	2	0	2	0
Previous medication				
Prednisone	7	0	13	11
Tacrolimus	1	0	0	0
MMF	1	0	1	1
Rituximab	0	7	0	14
Median time since RTX infusion (months, IQR)	N/A	9 (8.5-13.5)	N/A	5.5 (4-7.25)
Comorbidities				
Infectious				
URTI	5	2	0	0
Skin	2	0	0	0
Gastrointestinal	0	1	0	0
Inflammatory				
Airway (e.g. asthma)	1	1	0	0
Skin (e.g. eczema)	1	2	0	0

uPCR = urinary protein-to-creatinine ratio; SSNS = steroid-sensitive nephrotic syndrome; SRNS = steroid-resistant nephrotic syndrome; MCD = minimal change disease; FSGS = focal segmental glomerulosclerosis; MN = membranous nephropathy; MMF = mycophenolate mofetil; URTI = upper respiratory tract infection.
