# Supplementary material for: The extrafollicular B cell response is a hallmark of childhood idiopathic nephrotic syndrome
Source: Nat Commun. 2023 Nov 24;14:7682. doi: 10.1038/s41467-023-43504-8 (PMC10667257; doi:10.1038/s41467-023-43504-8)
Supplement: Supplementary file 3 — Description of Additional Supplementary Files [file 41467_2023_43504_MOESM3_ESM.pdf]

Description of Additional Supplementary Files

Supplementary Data 1. Participant characteristics. The demographic and clinical characteristics of all participants in this study.

Supplementary Data 2. Differences in gene expression between HC and INS PBMC lineages. The results of pseudobulk differential gene expression analysis between HC and INS for each PBMC lineage using the Monocle R package and the edgeR method. P_{adj} values were calculated using Benjamini-Hochberg corrections for multiple-testing.

Supplementary Data 3. Pathway analysis on the nephrotic B cell signature. The list of g:Profiler results showing the enrichment of Gene Ontology Biological Processes and Reactome pathways in the nephrotic B cell signature. P_{adj} (FDR) values were calculated using Benjamini-Hochberg corrections for multiple-testing.

Supplementary Data 4. Transcription factor enrichment analysis in the nephrotic B cell signature. The list of the ChEA3 results showing the ranking of predicted upstream regulators of the nephrotic B cell signature.

Supplementary Data 5. B cell subcluster gene expression. The results of differential gene expression analysis using Seurat comparing each B cell subcluster. P_{adj} values were calculated by Wilcoxon rank sum testing with Bonferroni correction.

Supplementary Data 6. Differentially expressed genes between HC and INS B cell subclusters. The results of pseudobulk differential gene expression analysis between HC and INS for each B cell subcluster using the Monocle R package and the edgeR method. P_{adj} values were calculated using Benjamini Hochberg corrections for multiple-testing.

Supplementary Data 7. Differences in gene expression between post-rituximab relapse and remission in memory and naïve B cells. The results of differential gene expression analysis using Seurat comparing post-rituximab relapse (Rel-RTX) and remission (Rem-RTX) memory and naïve B cells.
