# Supplementary material for: The extrafollicular B cell response is a hallmark of childhood idiopathic nephrotic syndrome
Source: Nat Commun. 2023 Nov 24;14:7682. doi: 10.1038/s41467-023-43504-8 (PMC10667257; doi:10.1038/s41467-023-43504-8)
Supplement: Supplementary file 11 — Reporting Summary [file 41467_2023_43504_MOESM11_ESM.pdf]

nature portfolio

Corresponding author(s): Dr. Tomoko Takano

Last updated by author(s): Oct 29, 2023

Reporting Summary

Nature Portfolio wishes to improve the reproducibility of the work that we publish. This form provides structure for consistency and transparency in reporting. For further information on Nature Portfolio policies, see our Editorial Policies and the Editorial Policy Checklist.

Statistics

For all statistical analyses, confirm that the following items are present in the figure legend, table legend, main text, or Methods section.

n/a	Confirmed	
☐	☒	The exact sample size (*n*) for each experimental group/condition, given as a discrete number and unit of measurement
☐	☒	A statement on whether measurements were taken from distinct samples or whether the same sample was measured repeatedly
☐	☒	The statistical test(s) used AND whether they are one- or two-sided<br>*Only common tests should be described solely by name; describe more complex techniques in the Methods section.*
☒	☐	A description of all covariates tested
☐	☒	A description of any assumptions or corrections, such as tests of normality and adjustment for multiple comparisons
☐	☒	A full description of the statistical parameters including central tendency (e.g. means) or other basic estimates (e.g. regression coefficient) AND variation (e.g. standard deviation) or associated estimates of uncertainty (e.g. confidence intervals)
☐	☒	For null hypothesis testing, the test statistic (e.g. *F*, *t*, *r*) with confidence intervals, effect sizes, degrees of freedom and *P* value noted<br>*Give P values as exact values whenever suitable.*
☒	☐	For Bayesian analysis, information on the choice of priors and Markov chain Monte Carlo settings
☒	☐	For hierarchical and complex designs, identification of the appropriate level for tests and full reporting of outcomes
☒	☐	Estimates of effect sizes (e.g. Cohen's *d*, Pearson's *r*), indicating how they were calculated

Our web collection on statistics for biologists contains articles on many of the points above.

Software and code

Policy information about availability of computer code

Data collection

FACSAria II (BD Biosciences)
LSRFortessa X-20 (BD Biosciences)
FACSDiva Software v8.0.1 (BD Biosciences)
10X Genomics Chromium Controller (10X Genomics)
NovaSeq6000 sequencer (Illumina)

Data analysis

For single-cell RNA-sequencing analysis:
CellRanger (v3.0.1)
R (v4.2.2)
Rstudio (v2022.07.0+548)
CellChat (v1.6.0)
circlize (v0.4.15)
cowplot (v1.1.1)
devtools (v2.4.5)
dittoSeq (v1.10.0)
DOSE (v3.24.2)
DoubletFinder (v2.0)
dplyr (v1.0.10)
edgeR (v3.40.0)
ggalluvial (v0.12.5)
ggplot2 (v3.4.0)

ggrepel (v0.9.2)
igraph (v1.3.5)
monocle3 (v1.3.1)
muscat (v1.14)
patchwork (v1.1.2)
RColorBrewer (v1.1-3)
reticulate (v1.26)
scater (v1.26.1)
SCENIC (v1.3.1)
Seurat (v4.3.0)

SeuratWrappers (v0.3.1)
stringr (v1.4.1)
tidyr (v1.2.1)
tidyverse (v1.3.2)
umap (v0.2.9.0)
uwot (v0.1.14)
VennDiagram (v1.7.3)

GSEA App (4.3.2)
Cytoscape (v3.9.1) with the EnrichmentMap, AutoAnnotate, and ClusterMaker2 plugins
g:Profiler (https://biit.cs.ut.ee/gprofiler/gost)
ChEA3 (https://maayanlab.cloud.chea3/)

For flow cytometry analysis:
FlowJo (v10.8) with the FlowSOM plugin
GraphPad Prism 9

All code used to analyze data and generate figures will be provided upon reasonable request.

For manuscripts utilizing custom algorithms or software that are central to the research but not yet described in published literature, software must be made available to editors and reviewers. We strongly encourage code deposition in a community repository (e.g. GitHub). See the Nature Portfolio guidelines for submitting code & software for further information.

Data

Policy information about availability of data

All manuscripts must include a data availability statement. This statement should provide the following information, where applicable:

- Accession codes, unique identifiers, or web links for publicly available datasets
- A description of any restrictions on data availability
- For clinical datasets or third party data, please ensure that the statement adheres to our policy

Raw sequencing data and the filtered count matrices were deposited in the NCBI Gene Expression Omnibus (GEO) as a super series under the accession number GSE233277. The results of all differential gene expression analyses, pathway analysis, and transcription factor analysis are included in Supplementary Data files. All source data needed to evaluate that conclusions are included with this paper. Public data repositories used for analysis include ChEA3 (https://maayanlab.cloud/chea3/) and g:Profiler (https://biit.cs.ut.ee/gprofiler/gost) for transcription factor enrichment and pathway analysis, respectively. The GRCh38 human reference genome was used for sequence alignment.

Research involving human participants, their data, or biological material

Policy information about studies with human participants or human data. See also policy information about sex, gender (identity/presentation), and sexual orientation and race, ethnicity and racism.

Reporting on sex and gender	Information regarding sex was collected from study participants and is reported in Supplementary Data 1 and Supplementary Table 1. However, the analyses performed did not consider sex or gender.
Reporting on race, ethnicity, or other socially relevant groupings	Information on race, ethnicity, or other socially relevant groupings were not collected from study participants.
Population characteristics	Relevant population characteristics are included in Supplementary Data 1. Information regarding age at sample collection, sex, urinary protein-to-creatinine ratio (uPCR), current medications, and related comorbidities (infection, atopy, etc.) were collected and reported.
Recruitment	For both healthy children and children with INS, parents/legal guardians were informed of the study and provided their written consent for blood collection and the use of the sample in our research by signing an informed consent form. In addition, children younger than 18 years of age and older than 7 years of age were informed of the study and were asked to sign an assent form. For children with INS, samples were collected either during hospital visits necessitated due to a proteinuric relapse or a scheduled visit while in remission (for follow-up or for treatment).<br><br>Blood samples were collected as part of a longitudinal observational cohort at the McGill University Health Centre (Montreal Children's Hospital) and the Alberta Children's Hospital. The populations of Montreal and Calgary are diverse, so we do not

	anticipate any selection biases in our recruitment approach. In the main group with active INS, we achieved equal numbers of males and females.
Ethics oversight	Children with idiopathic nephrotic syndrome (INS) were recruited according to protocols approved by the Research Institute of the McGill University Health Centre (REB: MUHC-14-466) and the Alberta Children's Hospital (REB: CHREB-16-2186). Healthy children (HC) were recruited at the Research Institute of the McGill University Health Centre (REB: MUHC-14-466). Blood samples from participants were used according with our standard operating protocol at the Research Institute of the McGill University Health Centre (SOP: MUHC-15-341).

Note that full information on the approval of the study protocol must also be provided in the manuscript.

Field-specific reporting

Please select the one below that is the best fit for your research. If you are not sure, read the appropriate sections before making your selection.

☒ Life sciences ☐ Behavioural & social sciences ☐ Ecological, evolutionary & environmental sciences

For a reference copy of the document with all sections, see nature.com/documents/nr-reporting-summary-flat.pdf

Life sciences study design

All studies must disclose on these points even when the disclosure is negative.

Sample size	Sample size was not pre-determined. Our sample size depended on sample recruitment at two sites, the McGill University Health Centre and the Alberta Children's Hospital as part of an ongoing national longitudinal cohort called CHILDNEPH. We used and reported on all samples that fit our criteria of relapse and remission. The sample sizes used in the single-cell RNA-sequencing (4 HC, 4 active INS, 4 Rel-RTX, 4 Rem-RTX) and immunophenotyping (24 HC, 13 active INS, 13 Rem-GC, 7 Rel-RTX, 14 Rem-RTX) studies are similar to those used in comparable studies. The lower sample number in the Rel-RTX group is reflective of the puacity of relapses taking place following RTX treatment.
Data exclusions	For single-cell RNA-sequencing studies, doublets were identified and removed using DoubletFinder, using the co-expression of lineage defining genes (CD79A, CD3G, CD14, LILRA4), and a feature threshold of no more than 3000 genes (HC-INS dataset) or 2500 genes (Rel-Rem dataset). Low-quality cells were identified as those with greater than 10% of UMIs mapping to mitochondrial genes, fewer than 7% mapping to ribosomal protein genes, expressing hemoglobin genes (HBA1, HBA2, and HBB) and removed from analysis. The results of this quality control are shown in Supplementary Figure 1.<br><br>No other data or participant was excluded from the study.
Replication	Single-cell RNA-sequencing studies were completed in four individuals per group (HC, INS, Rel-RTX, Rem-RTX). Replication was completed using three distinct clustering algorithms (reciprocal PCA integrated clustering, CCA integrated clustering, non-integrated clustering using Seurat) and differential gene expression analysis. Pseudobulk differential gene expression analysis was specifically reported to take into account biological replication. All attempts at replication of the reported data were successful in this regard.<br><br>The flow cytometry studies (FlowSOM panel in Figure 3 and atypical B cell phenotyping in FIgures 4 and 5) were completed independently. Biological replication was achieved by phenotyping 24 healthy controls, 13 individuals during active INS, 13 individuals in glucocorticoid-induced remission, 14 individuals in remission maintained by rituximab, and 7 individuals relapsing post-rituximab treatment. Technical replication was achieved given the redundancy of key markers in both flow cytometry panels that enable the identification of the major B cell subsets under study. There was no validation cohort available to verify these findings further.
Randomization	There was no randomization as groups were developed prior to immunophenotyping based on clinical history. Children were grouped into healthy (no diagnosis of INS or other kidney or immune-related diseases), active INS, post-RTX INS relapse, glucocorticoid-induced remission, and remission maintained by RTX using the criteria outlined in the "Human Subjects" category of the methods and as is reflected by Supplemental Data 1. All samples obtained during the study were used for our immunophenotyping analyses and reported in the manuscript.<br><br>For the single-cell RNA-sequencing study to compare PBMC from HC and INS individuals, we prioritized using samples from active INS individuals for whom we had obtained paired relapse-remission samples and represented multiple distinct stages of disease (first presentation, relapse, relapse on therapy) to ensure the robustness of our findings. HC were then selected by age/sex-matching to the active INS samples. For the single-cell RNA-sequencing study comparing total B cells from Rel-RTX and Rem-RTX, we selected 4 out of the 5 individuals for whom we had paired Rel-RTX/Rem-RTX samples by the availability of cells to ensure the feasibility of our studies.
Blinding	Individuals were de-identified and assigned a study identifier. Groupings were determined thereafter based on clinical parameters obtained from the physicians (uPCR, medication history). Thus, sample groupings were permanent (healthy, active INS, remission following glucocorticoids and/or rituximab). During the single-cell RNA-sequencing and flow cytometry experiments, researchers were blinded from sample groupings. Researchers were only made aware of the demographics and clinical history patients during data analysis for the purpose of data visualization.

Reporting for specific materials, systems and methods

We require information from authors about some types of materials, experimental systems and methods used in many studies. Here, indicate whether each material, system or method listed is relevant to your study. If you are not sure if a list item applies to your research, read the appropriate section before selecting a response.

Materials & experimental systems

n/a	Involved in the study
☐	☒ Antibodies
☒	☐ Eukaryotic cell lines
☒	☐ Palaeontology and archaeology
☒	☐ Animals and other organisms
☒	☐ Clinical data
☒	☐ Dual use research of concern
☒	☐ Plants

Methods

n/a	Involved in the study
☒	☐ ChIP-seq
☐	☒ Flow cytometry
☒	☐ MRI-based neuroimaging

Antibodies

Antibodies used

Anti-human CD3e BV785 (BioLegend, catalog 317330, clone OKT3, lot B360622) 1:50 dilution
Anti-human CD19 BV605 (BD Biosciences, catalog 562653, clone SJ25C1, lot 2129266) 1:20 dilution
Anti-human CD20 Alexa Fluor 700 (BioLegend, catalog 302322, clone 2H7, lot B368195) 1:50 dilution
Anti-human CD21 BV421 (BD Biosciences, catalog 562966, clone B-ly4, lot 2230272) 1:20 dilution
Anti-human CD27 PE-Cy7 (BD Biosciences, catalog 560609, clone M-T271, lot 1354575) 1:20 dilution
Anti-human CD10 BUV737 (BD Biosciences, catalog 612826, clone HI10a, lot 2122392) 1:20 dilution
Anti-human CD38 BUV737 (BD Biosciences, catalog 612824, clone HB7, lot 1320321) 1:20 dilution
Anti-human CD11c PerCp-Cy5.5 (BD Biosciences, catalog 565227, clone B-ly6, lot 2005722) 1:20 dilution
Anti-human IgD BV510 (BioLegend, catalog 348220, clone IA6-2, lot B354262) 1:20 dilution
Anti-human IgM Alexa Fluor 488 (BioLegend, catalog 314534, clone MHM-488, lot B340176) 1:40 dilution
Anti-human FcRL5 APC (BioLegend, catalog 340306, clone 509f6, lot B342168) 1:20 dilution
Anti-human CD24 PE (BD Biosciences, catalog 555428, clone ML5, lot 1167085) 1:20 dilution
Anti-human CD1c BV711 (BioLegend, catalog 331536, clone L161, lot B337734) 1:20 dilution
Anti-human CXCR5 APC (BioLegend, catalog 356908, clone J252D4, lot B326673) 1:20 dilution
Anti-human/mouse T-bet PE (BioLegend, catalog 644810, clone 4B10, lot B347807) 1:20 dilution
Anti-human/mouse Ki-67 BUV395 (BD Biosciences, catalog 564071, clone B56, lot 2341739) 1:50 dilution

Validation

The manufacturer validated all flow cytometry antibodies against isotype controls in human cells. We further validated the antibodies using fluorescence-minus-one or isotype controls to determine the optimal staining concentration in human PBMC (dilutions are reported above).

Flow Cytometry

Plots

Confirm that:

☒ The axis labels state the marker and fluorochrome used (e.g. CD4-FITC).

☒ The axis scales are clearly visible. Include numbers along axes only for bottom left plot of group (a 'group' is an analysis of identical markers).

☒ All plots are contour plots with outliers or pseudocolor plots.

☒ A numerical value for number of cells or percentage (with statistics) is provided.

Methodology

Sample preparation

PBMC were isolated from peripheral blood using a high density ficoll gradient. Isolated PBMC were cryopreserved in FBS supplemented with 10% DMSO until the immunophenotyping experiments were to be conducted. Cryovials containing PBMC were partially thawed in a 37C water bath and then emptied into tubes containing warm RPMI supplemented with FBS (10%), penicillin/streptomycin, gentamicin, sodium pyruvate, 1M HEPES, and non-essential amino acids (complete RPMI). Cells were pelleted, treated with DNase I, and washed with warm complete RPMI before resting for 2 hours at 37C in 5% CO2. Cells were then washed twice with cold PBS + 2% FBS and counted on a haemocytometer to ensure >90% viability and were then divided into a 96-well v-bottom plate for staining. No more than 1x10^6 cells were stained per flow cytometry panel (two panels). Cells were incubated with Fixable Viability eFluor780 Dye (ThermoFisher, catalog 65-0865-18) at 4C for 15 minutes, washed with PBS + 2% FBS, pelleted, and incubated with Fc receptor block (BD Biosciences, catalog 564219). Antibody cocktails for extracellular antibodies were prepared in PBS containing Brilliant Stain Buffer (BD Biosciences, catalog 563794) and added to the cells to achieve the proper dilution. Following a 20 minute incubation at 4C, cells were washed, and fixed using the Foxp3/Transcription Factor staining buffer set (eBioscience, catalog 00-5523-00). Following fixation, cells were washed in the 1X permeabilization buffer from the same set, and incubated with the antibody cocktail for intracellular markers for 45 minutes at 4C. Cells were then washed twice, once with 1X permeabilization buffer and a second time PBS + 2% FBS, before acquisition.

Instrument

BD LSRFortessa X-20

Software

FlowJo v10.8 software (Treestar) and Prism 9 (Graphpad)

Cell population abundance

B cells (CD19+ CD4- CD8a-) were FACS sorted before single-cell RNA-sequencing. The purity of this population was >99.5%. The proportions of immune cells evaluated in our immunophenotyping studies are shown in the data alongside sample flow cytometry plots. The source data contains proportions for each population investigated from every sample.

Gating strategy

Doublets were first excluded by gating on FSC-A/FSC-H and SSC-A/SSC-H. Lymphocytes were gated on by size and granularity (FSC-A/SSC-A) and dead cells were excluded as Viability eFluor780+ cells. Live cells were taken for further gating. For FlowSOM analysis, we used panel 1 which included the following markers: Viability eFluor780, CD3 BV785, CD19 BV605, CD20 Alexa Fluor 700, CD21 BV421, CD24 PE, CD27 PE-Cy7, CD38 BUV737, CD1c BV711, IgD BV510, IgM Alexa Fluor 488, CXCR5 APC, and Ki-67 BUV395. B cells were gated as CD19+CD3- cells from each individual before use for FlowSOM clustering. Following clustering, each generated metacluster was then assessed for expression of CXCR5, Ki-67, CD21, IgM, and IgD with positive gates being drawn from fluorescence-minus-one or isotype controls. For standard immunophenotyping aimed at characterizing CD21low B cells and defining them as atypical B cells, we used panel 2 which included the following markers: CD3 BV785, CD19 BV605, CD20 Alexa Fluor 700, CD21 BV421, CD27 PE-Cy7, CD10 BUV737, CD11c PerCp-Cy5.5, IgD BV510, IgM Alexa Fluor 488, FcRL5 APC, and T-bet PE. B cells were defined as CD19+CD3- and further divided into transitional naive B cells (CD10+CD20+), mature B cells (CD10-CD20+), and antibody-secreting cells (CD10-CD20-). Mature B cells were further divided into classical memory B cells (CD21+ CD27+), mature naive B cells (CD21+ CD27-), CD21low B cells (CD21low CD27+/-), atypical B cells (CD11c+ T-bet+). From each population, IgM+, IgD+, T-bet+, and FcRL5+ cells were gated based on fluorescence-minus-one and isotype controls. A sample gating strategy following the gating of CD19+ cells is shown in Figure 5b, but the complete gating strategy can be provided in the Supplementary information.

☒ Tick this box to confirm that a figure exemplifying the gating strategy is provided in the Supplementary Information.
